# 

**Published:** 1914-09

**Authors:** 


### NINETY-THIRD LIST OF BOOKS.

| | | | |
|---|---|---|---|
| **Chaplin, A.** | *Thomas Shortt* | | [1914] |
| **Cremach, J.** | *Chirurgische Diagnostik* | | 1914 |
| **Curgenven, J. S.** | *The Child's Diet* | 2nd Ed. | 1914 |
| **Elliot, R. H.** | *Sclero-Corneal Trephining in Glaucoma* | 2nd Ed. | 1914 |
| **Glaister and D. D. Logan, J.** | *Gas Poisoning in Industries* | | 1914 |
| **Hewat, A. F.** | *The Examination of the Urine* | 5th Ed. | 1914 |
| **Kenwood, H. R.** | *Public Health Laboratory Work* | 6th Ed. | 1914 |
| **Lane, Sir W. A.** | *Operative Treatment of Fractures* | 2nd Ed. | 1914 |
| **Logan, J. Glaister and D. D.** | *Gas Poisoning in Industries* | | 1914 |
| **Manson, Sir P.** | *Tropical Diseases* | 5th Ed. | 1914 |
| **Mercier, C. A.** | *Text-Book of Insanity* | 2nd Ed. | 1914 |
| **Millard, C. K.** | *The Vaccination Question in the light of Modern Experience* | | 1914 |
| **Pappenheim, A.** | *Clinical Examination of the Blood* (Translated by R. Donaldson) | | 1914 |
| **Rutherford, A. H.** | *The Ileo-Cæcal Valve* | | 1914 |
| **Short, A. R.** | *The Newer Physiology in Surgical and General Practice* | 3rd Ed. | 1914 |
| **Swietochowski, G. de** | *Mechano-Therapeutics* | | 1914 |
| **Weber, Sir H.** | *On Means for Prolongation of Life* | 4th Ed. | 1914 |
| **Whittaker, C. R.** | *Surgical Anatomy* | 2nd Ed. | 1914 |

#### TRANSACTIONS, REPORTS, JOURNALS, &c.

| | | |
|---|---|---|
| American Association of Obstetricians and Gynecologists, Transactions of the | Vol. XXVI | 1914 |
| American Journal of Obstetrics | Vols. LXVIII, LXIX | 1913–14 |
| American Journal of the Medical Sciences, The | Vol. CXLVII | 1914 |
| Archiv für klinische Chirurgie | Bd. CIII | 1914 |
| Archives of Pediatrics | Vol. XXX | 1913 |
| Boston Medical and Surgical Journal, The | Vols. CLXIX, CLXX | 1913–14 |

| | | |
|---|---|---|
| Bristol Health Report for 1913 .. .. .. .. .. .. .. .. .. .. | | 1914 |
| Bristol Medico-Chirurgical Journal, The .. .. .. .. | Vol. XXXI | 1913 |
| British Journal of Tuberculosis, The .. .. .. | Vol. VII | 1913 |
| British Medical Journal, The .. .. .. .. .. .. | Vol. I for | 1914 |
| Bulletin de la Académie royale de Médecine de Belgique .. .. | | 1913 |
| Bulletin de la Société française de Dermatologie .. .. .. .. .. | | 1913 |
| College of Physicians of Philadelphia, Transactions of the | Vol. XXXV | 1913 |
| Deutsche Zeitschrift für Chirurgie .. | Bds. CXXVI, CXXVII | 1914 |
| Edinburgh Medical Journal .. .. .. .. .. .. | N.S., Vol. XII | 1914 |
| Glasgow Medical Journal, The .. .. .. .. .. | Vol. LXXXI | 1914 |
| Hospital—St. Thomas's Hospital Reports.. .. .. | Vol. XLI | 1914 |
| Indian Medical Gazette, The .. .. .. .. .. | Vol. XLVIII | 1913 |
| International Abstract of Surgery .. .. .. .. | Vol. XVIII | 1914 |
| Journal of Experimental Medicine, The .. .. .. | Vol. XIX | 1914 |
| Journal of Hygiene .. .. .. .. .. .. .. .. .. | Vol. XIII | 1913–14 |
| Journal of Medical Research .. .. .. .. .. | Vol. XXIX | 1913–14 |
| Journal of Obstetrics and Gynæcology of the British Empire | Vol. XXIV | 1913 |
| Journal of the American Medical Association, The | Vol. LXII | 1914 |
| Lancet, The .. .. .. .. .. .. .. .. .. .. .. .. .. | Vol. I for | 1914 |
| Library Association Record, The .. .. .. .. .. | Vol. XV | 1913 |
| Library, The .. .. .. .. .. .. .. .. .. .. .. | 3rd Ser., Vol. IV | 1913 |
| Library World, The .. .. .. .. .. .. .. .. .. .. | Vol. XV | 1913 |
| Medical Chronicle, The .. .. .. .. .. .. .. .. | Vol. LVIII | 1913–14 |
| Medical Record .. .. .. .. .. .. .. .. .. .. .. | Vol. LXXXV | 1914 |
| Münchener medizinische Wochenschrift .. .. .. | Bd. I für | 1914 |
| Nordiskt medicinskt Arkiv .. .. .. .. .. .. .. .. .. .. .. .. | | 1912 |
| Parasitology .. .. .. .. .. .. .. .. .. .. .. .. .. .. | Vol. VI | 1914 |
| Philippine Journal of Science, The .. .. .. .. .. | Vol. VIII | 1913 |
| Practitioner, The .. .. .. .. .. .. .. .. .. .. .. .. | Vol. XCII | 1914 |
| Progressive Medicine .. .. .. .. .. .. .. .. .. .. | Vol. II | 1914 |
| Revue générale d'Ophtalmologie .. .. .. .. | Tom XXXII | 1913 |
| Surgery, Gynecology and Obstetrics .. .. .. .. .. | Vol. XVIII | 1914 |

### Obituary.

### Dr. CHARLES STEELE.

Charles Steele, M.D., F.R.C.S., who died at the age of 76, on Sept. 20th, at his residence, Richmond Hill, Clifton, was born in 1838, his father having been the Rev. John Steele, Rector of Christ Church, Macclesfield. After an early training which has cut short his career. We regret to hear that Mrs. Grace, to whom he had only been married twelve months, herself received a bullet wound.

---

### Local Medical Notes.

---

**University of Bristol.**—Students of the University have recently passed the following examinations:—

University of Bristol.—*M.B., Ch.B.—2nd Examination:* R. B. Britton, N. Durant, E. J. Ball, F. V. Jacques, R. H. Tasker; *Final Examination, Part I:* O. C. M. Davis. *B.D.S.—2nd Examination:* W. J. Langford. *Diploma in Dental Surgery — 2nd Examination:* R. L. O'Sullivan. *D.P.H.*—W. T. Torrance; *Part II:* J. R. Kay-Mouat.

University of London.—*M.B., B.S.:* H. J. D. Smythe, A. H. White; *2nd Examination, Part II:* F. K. Hayman; *Part I:* W. H. Royal.

D.P.H. Lond.—Capt. G. S. Parkinson.

Conjoint Examining Board of England.—*Practical Pharmacy:* R. I. Dacre, W. K. A. Richards. *Medicine:* A. G. Bodman, C. J. D. May. *Surgery:* C. N. Vaisey, R. I. Dacre, *C. J. D. May, — Foster. *Midwifery:* J. W. Gilbert, *R. I. Dacre, G. S. Terry.

L.S.A.—A. G. Bodman.

L.D.S.—*Chemistry:* S. K. Rigg, I. Jacobs. *Physics:* S. K. Rigg, A. J. Constance, B. Leathlean.

APPOINTMENTS.

Lt.-Col. P. G. Stock has been appointed Director of the South African Medical Service.

Henry Devine, M.D., B.S. Lond., M.D. Bristol, M.R.C.P., has been appointed Medical Superintendent of the Portsmouth Borough Asylum.

Walter Salisbury, M.B., B.S. Lond., has been appointed Resident Medical Officer at Queen Charlotte Lying-in Hospital, Marylebone.

* Denotes qualification.

